# Phytochemical and In Vitro Antibacterial Assessment of Selected Wild Edible Plants From Southwestern Ethiopia

**DOI:** 10.1155/ijfo/5113561

**Published:** 2026-07-09

**Authors:** Duresa Dedefo, Tamene Daba Rumicha, Gemmechu Hasen, Abdulgeni Mohammed, Diriba Taddese Legesse, Yetenayet Bekele Tola, Sirawdink Fikreyesus Forsido, Sileshi Belew, Sultan Suleman

**Affiliations:** ^1^ Department of Pharmacy, Hosanna College of Health Science, Hosanna, Central Ethiopia Regional State, Ethiopia; ^2^ Department of Food and Nutritional Sciences, Faculty of Agriculture, Wollega University, Shambu, Ethiopia, wollegauniversity.edu.et; ^3^ Department of Postharvest Management, College of Agriculture and Veterinary Medicine, Jimma University, Jimma, Ethiopia, ju.edu.et; ^4^ Clinical Trials Directorate, Armauer Hansen Research Institute, Addis Ababa, Ethiopia, ahri.gov.et; ^5^ Department of Internal Medicine, Grand Valley Primary Hospital, Jimma, Oromia, Ethiopia; ^6^ Department of Veterinary Microbiology, College of Agriculture and Veterinary Medicine, Jimma University, Jimma, Ethiopia, ju.edu.et; ^7^ Jimma University Laboratory of Drug Quality (JuLaDQ) and School of Pharmacy, Jimma University, Jimma, Oromia, Ethiopia, ju.edu.et

## Abstract

**Background:**

Wild edible plants (WEPs) are vital for food security and traditional medicine, particularly in resource‐limited areas. Despite their potential, their use is constrained by a lack of scientific evidence. This study assesses the phytochemical properties and antibacterial activity of chosen WEPs from southwestern Ethiopia.

**Method:**

Observational and experimental study designs were utilized to identify and select WEPs through focus group discussions, key informant interviews, and multicriteria decision analysis. WEP samples were collected, dried, powdered, and extracted separately using methanol, ethanol, and acetone solvents. Physicochemical and qualitative phytochemical screenings were evaluated following standardized procedures. Antibacterial activity was evaluated using agar disk diffusion and broth dilution methods against *Staphylococcus aureus* and *Escherichia coli*, with concentrations varying from 10 to 100 mg/mL. Gentamicin served as a positive control, whereas 2% dimethyl sulfoxide (DMSO) acted as a negative control. Data analysis was performed using Microsoft Excel 2010 and R software (Version 4.5.2). A three‐way ANOVA analyzed the effects of solvent type, bacterial species, and extract concentration; Tukey′s HSD for post hoc was applied for comparisons at *α* = 0.05 to denote statistically significant differences.

**Results:**

Among 22 medicinally important WEPs, *Mussaenda arcuata* and *Pteridium aquilinum* were selected for experimentation. *M. arcuata* exhibited higher water and ethanol extractive values (*p* < 0.001). Alkaloids, glycosides, tannins, flavonoids, saponins, steroids, phenols, terpenoids, quinones, protein, amino acids, and lipids or oils were observed phytochemical compounds, with the exception that *P. aquilinum* lacked reducing sugars. The acetone extract of *M. arcuata* demonstrated significant antibacterial activity against *S. aureus* at 100 mg/mL, with a zone of inhibition of 17.2 ± 0.11 mm (*p* < 0.05), surpassing the ethanol extract′s 16.2 ± 0.07 mm. Both ethanol and acetone extracts effectively inhibited the growth of *S. aureus* and *E. coli*, establishing a minimum inhibitory concentration of 25 mg/mL.

**Conclusion:**

The extracts of *M. arcuata* exhibited a dose‐dependent antibacterial activity against both *S. aureus* and *E. coli* bacteria, with the acetone extract proving more potent than the ethanol extract. *M. arcuata* is recommended for further research on its active compounds and safety for potential antimicrobial development, whereas *P. aquilinum* showed no significant antibacterial activity despite the presence of bioactive compounds.

## 1. Introduction

Wild edible plants with medicinal properties play a critical role in food security and community health by tackling malnutrition and treating various health problems globally [1, 2]. They have a therapeutic potential in preventing and treating numerous chronic diseases and improving physical fitness, as well as boosting overall vitality and regaining good health [3–5]. Despite their importance, these naturally growing and reproducing plant species have been significantly neglected from a scientific standpoint for a long time, with only 10% of medicinal plants currently cultivated, with the majority remaining wild [6–8].

WEPs provide edible parts such as fruits, leaves, seeds, shoots, roots, and tubers that are rich in nutrients and bioactive compounds [9]. They possess bioactive chemicals that are used as a potential source of functional foods and traditional medicine [10]. These plants provide crucial, affordable, profitable, and nutritionally significant contents such as essential vitamins, antioxidants, fiber, minerals, essential fatty acids, proteins, and other nutrients, which are vital, particularly for economically unstable populations facing food insecurity [11]. Unlike cultivated or highly processed foods, which may be contaminated with harmful chemicals and pesticides, WEPs offer a safer alternative and exhibit stronger antibacterial properties against certain pathogens [12, 13].

Globally, at least one billion people consume WEPs for food and medicine, according to the Food and Agriculture Organization [14]. The World Health Organization (WHO) report states that around 80% of people in the world use some form of traditional, complementary, and alternative medicine, usually alongside modern Western‐style healthcare services [15]. This dependence highlights the critical role of WEPs in addressing the food–medicine continuum [16], with more than 20,000 plant species consumed medicinally in diverse human cultures [17]. Consequently, many researchers are becoming more interested in examining the overlap between nutritional and medicinal applications of WEPs in several social‐ecological systems.

For example, consumption of WEPs has been a part of traditional European medicine and human nutrition; hence, extensive scientific research on these plants has been conducted in the continent for a variety of reasons. First, primary nutritional constituents (proteins, lipids, carbohydrates, vitamins, and minerals) are known to be abundant in edible wild plants. Second, many biologically active components in WEPs have been shown to have positive health effects and potential usefulness as feed additives, nutritional supplements, and therapeutic agents. Third, a vast genetic resource from WEPs can be utilized in breeding programs to improve the nutritional and pharmacological value of cultivated plants as well as to make them more resistant [18–20].

Additionally, the study in a coastal region of South China revealed the importance of food–medicine plants for chronic disease treatment and nutrition purposes, especially in developing and minority groups [21]. The study conducted in Northeastern Brazil on WEPs and the food–medicine continuum demonstrated that the primary medicinal uses of salient species of *Anredera cordifolia* were linked to intestinal regulation, stomach problems, and body strengthening [16]. The study carried out in Lebanon revealed that people believed that eating wild foods could cure the majority of human illnesses [22]. When compared with the findings from other nations, it is found that 12 of them are only consumable in Yemen from 58 wild plant species that were used as food for the first time, whereas 46 are consumable in several countries, primarily in East Africa [23]. According to a study carried out in Northeast India, the plants *Natsiatum herpeticum* and *Sphenoclea zeylanica* are known to contain minerals and a variety of bioactive chemicals that offer several health benefits upon consumption [24]. As evidenced by a 1975 survey conducted in Mozambique, WEPs were suggested as essential African diets [25]. Food scarcity, the spiciness of staple foods and their therapeutic and nutritional value, and cultural practices are the reasons given for the wide consumption of WEPs, according to a study in Uganda [26].

Ethiopia ranks fifth in tropical Africa and among the Top 25 richest countries globally in plant diversity [27]. The Ethiopian population possesses vast knowledge, cultures, and opportunities for utilizing WEPs [28]. Actually, around 30%–40% of the Ethiopian population were main consumers of WEPs, and this percentage was even higher, reaching up to 56%–67% in some areas in the country [29]. It has been documented that 413 WEPs from 77 families, 224 genera, and around 6000 species were collected in total, of which 10% were the indigenous ones [30, 31]. Approximately 70% of WEP has been utilized as folk medicine to treat various medical disorders [17]. WEPs were used as a survival strategy during intensified food shortages due to low agricultural development and lack of rain, highlighting the persistent problem of food insecurity in different parts of Ethiopia [17, 32]. For example, the Konso ethnic group in southern Ethiopia survived by consuming WEPs for three severe drought seasons from 1996 to 1999 [33].

WEPs such as *Tamarindus indica*, *Ximenia americana*, *Ziziphus spina-christi* Willd., *Adansonia digitata* L., *Asparagus africanus*, *Cordia africana*, and *Carissa spinarum* have been traditionally used by different Ethiopian ethnic groups [34–37]. A study from Nech Sar National Park revealed that 18 WEPs were used as a source of traditional medicine and food by the local communities [38].

The WHO, referenced by Annan et al., states that physicochemical and phytochemical investigations should be performed to confirm the identity, purity, and quality of plants or their preparations to ensure reproducible quality [39]. One of the essential factors for plants′ medicinal properties is the presence of very important phytochemicals like alkaloids, tannins, flavonoids, saponins, glycosides, phenolics, steroids, terpenoids, and reducing sugars, which are the source of a wide range of medicinal properties, whether they are used separately or in combination [40, 41].

WEPs have also recently gained attention and are a great option for treating infectious diseases that become more resistant due to antimicrobial resistance (AMR) [42]. The development of AMR in common pathogens makes routine medical procedures increasingly dangerous and reduces the effectiveness of standard treatments [43]. Currently, at least 700,000 people die each year due to AMR globally [44]. Estimation suggests that AMR could cause 10 million deaths that have economic losses anticipated to exceed 100 trillion USD annually by 2050 [45, 46]. This crisis primarily affects developing countries, where AMR could force up to 24 million people into extreme poverty by 2030 [47]. Patients with antibiotic‐resistant infections tend to have higher death rates and require more hospitalization days in comparison with those with susceptible infections [48]. So, a worldwide collaboration, including stewardship programs, better surveillance mechanisms, and innovative antimicrobial agents, is necessary to prevent the development of AMR bacteria that are increasing quickly [49]. Meanwhile, active exploration of WEP as a potential treatment of infectious diseases is significant since many of these compounds are highly effective in combating microbial pathogens currently [50–52]. These provide urgent context for WEP research.

Numerous studies on medicinal plants in Ethiopia have demonstrated promising antibacterial activities. For example, *Acokanthera schimperi* and *Brucea antidysenterica* inhibited the growth of the test organisms by 100% and 35%, respectively [53]. A recently conducted study showed that *Thalictrum rhynchocarpum* demonstrated outstanding activity against *Staphylococcus aureus* and *Escherichia coli* with minimum inhibitory concentrations (MICs) as low as 0.48 *μ*g/mL [54]. *Polystachya steudneri* Rchb.f.′s ethanol‐based extract demonstrated potency against *S. aureus* and *E. coli*, with MIC values of 8 and 3 mg/mL, respectively [55]. Hence, it becomes rational to use in vitro antibacterial activity assessment to explore specific pathogens′ susceptibility, sensitivity, and resistance to various chemical constituents extracted from medicinally useful WEPs [56].

Despite the strong traditional knowledge of WEPs in Ethiopia [17, 57, 58], scientific evidence supporting their use has been neglected and underestimated for many years [59]. The relationship between the traditional knowledge of WEPs and their potential as nutraceuticals from a modern scientific standpoint is also limited. Moreover, the medicinal uses of major WEPs have not yet been assessed scientifically in different parts of Ethiopia, even though some efforts have been made to identify and characterize their profiles using a traditional ethnobotanical survey [30, 60]. Therefore, the current study is aimed at investigating the underexplored area of southwestern Ethiopia, an area rich with biodiversity, but previously underdocumented for WEPs with their potential medicinal activities. To ensure scientific rigor, a direct rating‐based multicriteria decision analysis (MCDA) approach was utilized to prioritize WEPs for laboratory experimentation. This method provides a transparent and reproducible alternative to traditional selection processes, offering significant utility for ethnobotanical and nutraceutical investigations [61]. Implementing scientifically proven WEPs may solve problems of increased incidences of chronic noncommunicable diseases that could result from unhealthy conventional diets [14, 62]. Moreover, a new empirical data from phytochemical and antibacterial evaluations of WEPs could support the discovery of antibacterial medicines, potentially addressing the rise of global AMR bacteria [63]. If proven effective, WEPs may provide a low‐cost, accessible alternative to conventional antibiotics in resource‐limited areas, reducing dependence on synthetic drugs and minimizing AMR side effects. Thus, the current study is aimed at bridging this gap by conducting phytochemical and in vitro antibacterial activity assessments of selected WEPs from southwestern Ethiopia.

## 2. Materials and Methods

### 2.1. Study Area

**Figure 1:**
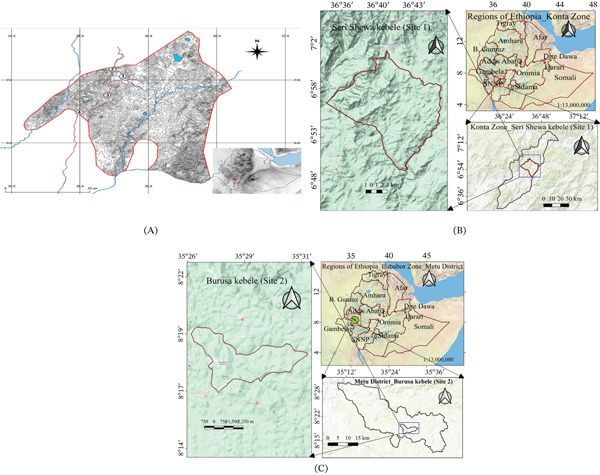
(A) Map of Chebera Churchura National Park (adapted from Martynov et al.) (B) Map of Seri Shewa kebele (C) Map of Burusa kebele.

The plant materials were collected from two purposively selected study areas in southwestern Ethiopia. The southwestern portion of Ethiopia covers more than 62.5% of the forest areas in the country, which have been used as a source of traditional medicine to treat various human and livestock diseases [6]. Seri Shewa kebele, located around Chebera Churchura National Park (CCNP), was the first study area. This area is located at the center of the Omo‐Gibe River Basin, in Konta Special Woreda, Konta Zone, along the southwestern region of Ethiopia, 480 km south of Addis Ababa. It is located at 1700 m above sea level and covers an area of 1250 km^2^. The CCNP is extremely rich in natural vegetation like riverine forests, savannas, predominant elephant grass, and montane evergreen forests [64, 65]. Burusa kebele, located in the Metu district, Ilu Aba Bor Zone, in the Oromia region, was the second study area [66]. This study area is one of the rainy areas with a high proportion of rainfall in the country [67]. Maps of study areas are presented in Figure 1.

### 2.2. Study Period and Design

The study employed a mixed method approach, combining observational surveys with an experimental design. The desk review meeting with the data collection team was held on November 17, 2023, to determine the study area and design selection criteria for the MCDA for candidate WEP species. Purposively, a preliminary observational survey was conducted to identify medicinally useful WEPs in selected study areas from April 1 to 30, 2024. Focus group discussions and key informant interviews were used to identify and collect data on medicinally important WEP species. A focus group consisting of small groups of participants discussed a specific topic under the moderator′s guidance. It includes a preliminary survey to identify key themes, followed by a focus group discussion to explore these themes in depth [68]. In this study, a focus group discussion and key informant interview on details of each WEP species regarding their local names, parts used, habits (mode of growth), habitats, medicinal uses, collection modes, preparation modes, consumption modes, harmful effects, and seasonal availabilities were employed. Besides, multiphased experimental approaches were used to characterize the physicochemical, phytochemical, and in vitro antibacterial activities of selected WEPs.

### 2.3. Selection of WEPs Using MCDA

The study utilized a direct rating‐based MCDA approach to establish a transparent and objective selection process of WEPs suitable for laboratory experimentation. This framework assesses and prioritizes multiple conflicting factors, promoting logical and evidence‐based decision‐making in complex scenarios [69, 70]. Since the 1960s, this approach has increasingly attracted attention across various disciplines [71]. Accordingly, a comprehensive desk review was conducted by a panel of interdisciplinary decision‐makers at Jimma University to identify and determine the key selection criteria for prioritizing WEPs. Through consensus, 10 critical criteria were decided by assigning equal weight to each criterion in the selection process. Finally, the two highest‐scoring WEP species were selected for further physicochemical, phytochemical, and antibacterial investigations to make the study feasible and specific. The details of the selection criteria and total score of each WEP are depicted in File S1.

### 2.4. Description of the Selected WEP

Based on an MCDA approach, two WEPs were selected from a total of 22 identified plant species. They were the leading candidates for experimental investigation, each scoring 9 out of 10 criteria (File S1, Table 1). These WEPs were *Mussaenda arcuata* (locally named Mentserqo) from Seri Shewa kebele and *Pteridium aquilinum* (locally named Gixo) from Burusa kebele. Their study parts (fruit and fiddlehead) have been consumed as food and as folk medicine by local communities to treat various medical disorders, including infectious diseases like diarrhea, wound infections, and related symptoms.

**Table 1:** Ethnobotanical study of medicinally important WEPs identified at Seri Shewa and Burusa kebeles.

Local name	Scientific name	Family	Part used	Growth habit	Habitat	Medicinal use	Collection mode	Preparation mode	Consumption mode	Seasonal availability
Haram Gusa^O^	*Ajuga integrifolia*	Lamiaceae	Leaf	Herb	Moist ground and shaded grassy slopes	BP and back pain	Cut	Cook	Eaten	Evergreen
Gixo^O^	*Pteridium aquilinum*	Dennstaedtiaceae	Fiddle head	Herb	Moist and acidic soils, open woodlands, and grasslands	Wound and diarrhea	Cut	Boil	Eaten	Evergreen
Amasillo^O^	*Celosia trigyna* L.	Amaranthaceae	Leaf	Herb	Mountainous region, roadsides, short grassland, damp areas	Parasite tapeworm	Cut	Cook	Eaten	Evergreen
Qoricha barilee^O^	*Rumex nervosus* Vahl	Polygonaceae	Leaf	Herb	Wetlands, swamps, and the banks of streams and rivers	Diarrhea	Cut	Boil	Eaten	Perennial
Bosoqqe^O^ (Indahula^A^)	*Kalanchoe petitiana* A.Rich	Crassulaceae	Leaf	Herb	Mountainous to woodlands	Tumor and evil eye	Cut	Grind	Apply to the tumor	Perennial
Obxii^O^	*Solanum nigrum* L.	Solanaceae	Leaf	Herb	Disturbed habitats, roadsides, and fields	Abdominal pain	Cut	Boil	Eaten	Rainy season
Haanquu^O^	*Embelia schimperi* Vatke	Primulaceae	Leaf	Tree/shrub	Upland thickets, primary forest, jungle edges, and roadsides	Tapeworm	Cut	Boil	Eaten	Evergreen
Agamsa^O^ Agam^K^	*Carissa spinarum* (Vahl.)	Apocynaceae	Fruit	Shrub	Dry deciduous forests, coastal thickets, and disturbed habitats	Parasite	Raw	Boil	Drunk	Evergreen
Rumanii^O^	*Punica granatum*	Lythraceae	Fruit	Tree	Sunny climates and often found in dry limestone soils	Diabetes	Raw	Ripe	Eaten	Dry season
Bakkanisa^O^	*Croton macrostachyus* H.	Euphorbiaceae	Leaf	Tree	Rocky hillsides, gully forests, and swamp forests	Parasite	Cut	Cook	Smell	Evergreen
Burii^O^	*Impatiens rothii* Hook.f.	Balsaminaceae	Root	Herb	Damp rainforests, shrubby sites, ravines, stream margins, grassy slopes	Blood pressure	Remove	Cook	Eaten	Rainy season
Goraa^O^	*Rubus apetalus*	Rosaceae	Fruit	Shrub	Living fences and roadsides	Anemia	Ripe	Ripe	Eaten	Evergreen
Qaqawwi^O^	*Rosa abyssinica* L.	Rosaceae	Fruit	Herb	Dry deciduous forests, woodlands, and scrublands	Diabetes	Raw	Ripe	Eaten	Perennial
Hawwixii^O^	*Physalis peruviana* L.	Solanaceae	Fruit	Herb	Disturbed habitats, gardens, and agricultural areas	Dyspepsia	Raw	Ripe	Eaten	Rainy season
Baddeessaa^O^	*Syzygium* G	Myrtaceae	Fruit	Tree	Forests	Anthelmintic	Raw	Ripe	Eaten	Evergreen
Waddeessa^O^	*Cordia africana* Lam.	Boraginaceae	Fruit	Tree	Dry deciduous forests, woodlands, and scrublands	Stomach aches	Raw	Ripe	Eaten	Evergreen
Meexxii^O^	*Phoenix reclinate*	Arecaceae	Fruit	Tree	Riverbanks, savannas, rocky hillsides, and grasslands	Digestive aid	Raw	Ripe	Eaten	Evergreen
Kurawa^O^ Koshim^K^	*Dovyalis abyssinica*	Flacourtiaceae	Fruit	Tree	Rain/riparian forest, scrublands, wooded grassland, deciduous bushland, and rocky limestone	Stomach ulcer	Ripe	Ripe	Eaten	Evergreen
Kussayee^O^ Kushita^K^	*Lippia adoensis* Hochst.	Verbenaceae	Leaf	Herb	Dry savannas, woodlands, and disturbed areas	Internal parasite	Raw	Boil	Eaten	Evergreen
Kosorruu^O^	*Acanthus sennii*	Acanthaceae	Leaf	Shrub	Dry forests, secondary scrub, and grasslands	Wound healing	Cut	Grind	Apply to the wound	Perennial
Mentserqo^K^	*Mussaenda arcuata*	Rubiaceae	Fruit	Shrub	Woodland forests, shaded riverine areas, rocky outcrops, roadsides, savannas, and grasslands	Internal parasite	Raw	Ripe	Eaten	Evergreen
Kore^K^	*Sporobolus pyramidalis* P.	Poaceae	Fruit	Shrub	Dry, sandy soils in open woods, prairies, and savannas	Diarrhea	Raw	Ripe	Drink	Perennial

*Note:* The uppercase superscript letters indicate the following: O = Afan Oromo language, A = Amharic language, and K = Konta language.

Abbreviations: BP, blood pressure; NR, not reported.

### 2.5. Study Variables

The study identified the following independent and dependent variables:

#### 2.5.1. Independent Variables

The independent variables include the indigenous knowledge and practice on WEP, selection criteria for WEPs based on the MCDA approach, types of solvent used for extraction, concentrations of extracts, and bacterial strains used.

#### 2.5.2. Dependent Variables

The dependent variables include the physicochemical properties of selected WEPs, the presence or absence of phytochemical constituents in selected WEPs, and in vitro antibacterial activities of selected WEPs.

#### 2.5.3. Control Variables

The variables kept constant throughout the course of an experiment to ensure a fair test were inoculum concentration, incubation time and temperature, solvent residues (to ensure complete evaporation of solvents), and media type (Mueller–Hinton agar [MHA]).

### 2.6. Selection of Key Informants and Focus Group Discussion

**Figure 2:**
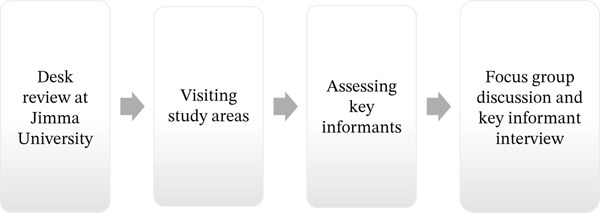
Steps followed for the selection of key informants in the study areas.

Key informants in study areas were purposively selected based on the standard method, and the brief selection procedure is depicted in Figure 2. The key informants were selected with the help of local administrators and community leaders from both study areas. The key informants in those areas were contacted and interviewed to identify the medicinally important WEPs. Focus group discussions were performed with selected key informants. The medicinally important WEPs data were collected from key informants through a semistructured questionnaire. Group discussions facilitated by a moderator were conducted to obtain details of the WEPs, plant parts used, when to use, why to use, how to use, how to prepare, where to collect from, when to collect, and harmful effects.

### 2.7. Field Trips for Identification of Selected WEPs

Field walks were conducted with local people or selected key informants to observe, identify, and collect WEPs. Senior researchers provided technical support to validate the WEPs during the survey.

### 2.8. Collection of Selected WEPs

The identified and selected WEP samples were collected and transported to Jimma University′s postharvest management and animal science laboratories to determine their physicochemical characteristics, screen their phytochemical constituents, and assess their in vitro antibacterial activities.

### 2.9. Experimental Procedure

#### 2.9.1. Preparation of Crude Plant Extracts

The healthy, fresh samples of collected WEPs were thoroughly washed manually using distilled water. Then, they were dried at room temperature to prevent the deterioration of secondary metabolites available in the plant materials. The dried plant materials were ground into a fine powder using the mortar and pestle. The ground plant samples were stored in clean and tightly closed plain polythene bags and bottle containers for further investigations.

#### 2.9.2. Extraction of Plant Material

Continuous shaking and maceration extraction procedures were done following the standard guidelines [72, 73] by using the solvents, including acetone (99.5%), ethanol (99%), and methanol (99.8%). The solvents were removed after extraction using a rotary evaporator at different temperature ranges.

##### 2.9.2.1. Sample Preparation for Phytochemical Screenings

Ten grams of dried and coarsely ground powder samples of each WEP was mixed with 100 mL of methanol solvent (at 1 g powder sample in 10 mL solvent standard proportion) in Erlenmeyer flasks. Extractions of samples were carried out by using a shaker (HY‐2A, speed‐adjusting multipurpose mechanical shaker, China). The mixture was shaken at a speed of 150 rpm for 24 h at room temperature and then filtered using Whatman filter paper. The filtration process was repeated three times to extract significant amounts of the chemical constituents from the plant materials. The final extracts were stored in a refrigerator at 4°C for phytochemical screening.

##### 2.9.2.2. Sample Preparation for In Vitro Antibacterial Activity Assessment

**Figure 3:**
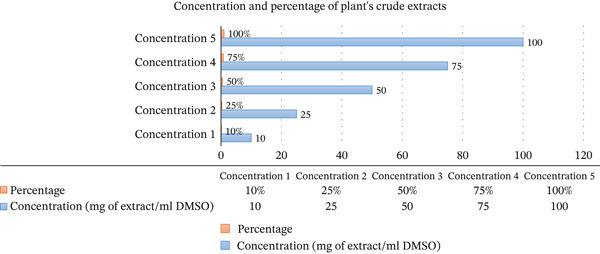
Concentrations of crude extracts used for the in vitro antibacterial activity test.

Plant crude extracts for an in vitro antibacterial activity assessment were prepared by mixing air‐dried, powdered plant samples separately with ethanol and acetone solvents in labeled conical flasks. A sample‐to‐solvent ratio of 1:10 (w/v) was used to provide optimal solubilization of plant constituents. The mixtures were filtered three times with Whatman filter paper after they had been shaken on a mechanical shaker for 72 h at room temperature. After extraction, the solvents were removed using a rotary evaporator to obtain concentrated crude extracts. The desiccator was used to dry crude extracts, and a metal mantle was used to gently heat them to eliminate any remaining residual solvents. The concentrated crude extracts were dissolved in 2% DMSO to achieve a 0.1 g/mL final concentration. Five working concentrations were then created, ranging from 10 to 100 mg/mL (Figure 3). The 2% DMSO served as a nontoxic cosolvent to ensure the solubility of both polar and nonpolar bioactive constituents for use in agar disk diffusion and broth dilution assays. The extracts were stored in a refrigerator at 4°C to be used for antibacterial assessments.

#### 2.9.3. Physicochemical Test

Physicochemical parameters like water‐extractable and ethanol‐extractable matter were determined according to WHO standard guidelines [74, 75]. Briefly, water‐ and ethanol‐extractable matter were determined as follows: 4‐g powder of each plant material was placed in a glass‐stoppered conical flask. One hundred milliliters of distilled water and ethanol was added to the separate flasks and weighed to obtain the total weight, including the flasks. The flasks were then shaken adequately and allowed to stand for an hour. A reflux condenser was fitted to the flasks, which were slowly boiled for 1 h before being cooled and weighed. The weight was readjusted to the original total weight by adding the required amount of distilled water. The flask was shaken adequately and rapidly filtered using dry filter paper. Then, 25 mL of the filtrate was transferred to a tarred flat‐bottomed dish and evaporated in a water bath until dry. Then, the dish was dried at 105°C for 6 h and weighed after cooling in a desiccator. The content of extractable matter of the materials was calculated using the following formula:

%Water/ethanol−extractable matter=weight of residueweight of sample∗4100∗.

#### 2.9.4. Phytochemical Screenings

The qualitative phytochemical screenings were conducted using standard procedures for testing the presence or absence of biologically active phytochemical compounds [76–78]. Accordingly, the standard test methods including alkaloid screening by Wagner′s test, glycoside screening by Liebermann′s test, tannin screening by Braymer′s test, flavonoid screening by alkaline reagent test, saponin screening by foam test, steroid screening by the Salkowski test, phenol screening by the ferric chloride test, terpenoid screening by concentrated H_2_SO_4_ test, quinones screening using concentrated HCl, reducing sugar screening by Benedict′s test, protein screening by Biuret test, amino acid screening by Xanthoproteic test, and lipid or oil screening by spot test were used for qualitative detection of various phytochemical groups.

#### 2.9.5. In Vitro Antibacterial Activity Assessment

Two clinically significant and representative bacterial strains, Gram‐positive *S. aureus* (ATCC 29213) and Gram‐negative *E. coli* (ATCC 25922), were used for in vitro antibacterial activity assessments due to their prevalence in causing infections, antibiotic resistance profiles, and distinct cell wall structures representing their respective Gram categories [79–82]. Gentamicin disk (10 *μ*g/mL) served as a positive control, demonstrating significant inhibition of both bacteria [83], whereas DMSO (2%) was used as the final solvent of plant material and served as a negative control due to its inert nature without interfering with the antibacterial activity of plant materials under test [84, 85]. Antibacterial activity was assessed using the agar disk diffusion and broth dilution methods following established standard procedures and previous studies [86, 87]. Briefly, for agar disk diffusion, an MHA was prepared by mixing agar media with distilled water [88], autoclaving at 121°C for 15 min, and solidifying in sterile petri dishes. The inoculum was adjusted to the 0.5 McFarland turbidity standards (1.5 × 10^8^ CFU/mL) by suspending three comparable colonies from each bacterium in 5 mL of MHA. The bacterial suspension was swabbed onto the agar, and filter paper disks immersed in varying concentrations of plant extract (100, 75, 50, 25, and 10 mg/mL) were placed on the surface. After 2 h at room temperature, the zones of inhibition (ZOIs) were measured in millimeters. Moreover, for quantitative determination, the MIC was established using the broth dilution method. Serial dilutions of each extract were prepared under sterile conditions, thoroughly mixed, and aseptically applied to MHA plates inoculated with the respective bacterial suspension. Following incubation at 37°C for 48 h, the MIC was defined as the lowest concentration of the extract that showed complete inhibition of visible bacterial growth.

### 2.10. Statistical Analysis

The data were analyzed qualitatively and quantitatively by organizing all quantitative information from key informants prior to analysis. Microsoft Excel 2010 was used for managing spreadsheets and employed data visualization tools like figures and tables to illustrate key results and aid interpretation. All experiments were performed in triplicate (*n* = 3), with results presented as mean ± standard deviation. Besides, the physicochemical was analyzed to evaluate the effects of plant species and solvent type. For antibacterial activity, a three‐way ANOVA was conducted to assess the effects of solvent type, bacterial species, and extract concentration. For a significant *F*‐test (*p* < 0.05), Tukey′s HSD test was used for post hoc comparisons, with significance set at *α* = 0.05 and results annotated with a letter‐based superscript system to indicate statistically significant differences. In both ANOVA, R software (Version 4.5.2) was utilized.

### 2.11. Materials and Reagents

The study used common laboratory material/equipment which included aluminum foil papers, autoclave, beakers, bottles, Bunsen burners, capillary tubes, conical flasks, cotton wool, crucibles, desiccator, evaporator, filter paper, forceps/tongs, funnels, glass funnels, glass stirrers, hand gloves, incubators, measuring cylinders, metal mantle, meter rulers, microtiter plates, mortars and pestles, multipurpose mechanical shaker, multichannel micropipette, ovens, petri dishes, pH meters, polythene bags, refrigerators, rotary muffle furnace, spatulas, stainless steel trays, test tubes, test‐tube racks, thermometers, thermostat water bath, top mettled weighing balance, weighing scales, wire gauze, and vinyl tubes. The chemicals or reagents used in this study consisted of the following analytical‐grade substances, including 99.8% methanol (Loba Chemie Pvt. Ltd), 99.5% acetone (Indenta Pvt. Ltd, India), 99% ethanol (Yedno Industry PLC), sodium hydroxide (Loba Chemie Pvt. Ltd), hydrochloric acid (Loba Chemie Pvt. Ltd), sulfuric acid (Loba Chemie Pvt. Ltd)_,_ ferric chloride (Loba Chemie Pvt. Ltd), potassium hydroxide (Loba Chemie Pvt. Ltd), distilled water (Jourilabs), chloroform (Alpha Chemika), copper sulfate (Guangdong Guanghua Sci‐Tech Co. Ltd), nitric acid (Loba Chemie Pvt. Ltd), Wagner′s solution (Jimma University Laboratory of Drug Quality), Benedict′s reagent (Loba Chemie Pvt. Ltd), bacterial strains (obtained from Armauer Hansen Research Institute [AHRI]), 0.5 McFarland standard (obtained from AHRI), and MHA media (obtained from AHRI). To ensure their reliability and purity in experimental procedures, all chemicals/reagents were handled according to the standard set by laboratory safety protocol by sourcing from reputable suppliers.

## 3. Results

### 3.1. Ethnobotanical Characteristics

A total of 22 medicinally important WEPs used by local communities for the treatment of various health conditions were identified and documented. Seventeen plant species were reported from Burusa kebele, two from Seri Shewa kebele, and three from both. Fruits were the predominant WEPs used, comprising 50% of reported uses. *M. arcuata* was used for the treatment of internal parasites and gastrointestinal disorders, whereas *Punica granatum* was used for diabetes, and *Rubus apetalus* for anemia. Leaves were the second most commonly used, accounting for 40.9% of uses, with *Ajuga integrifolia* for hypertension and *Kalanchoe petitiana* for tumor treatment. Other parts like roots and stems were less frequently cited but remain essential for traditional medicine. Besides, herbs were the most common growth habits of documented WEPs, comprising 40.9%, followed by trees at 22.7%. No harmful effects are reported in all identified WEPs. A comprehensive summary of medicinally important WEPs, including key details such as their local name, scientific name, family, plant parts used, growth habits, habitats, traditional medicinal uses of the identified WEPs based on the reports by key informants, mode of collection, mode of preparation, mode of consumption, and seasonal availability, is presented in Table 1.

### 3.2. Physicochemical Analysis

A two‐way ANOVA revealed that plant species (*F* = 436.18, *p* < 0.001) and solvent type (*F* = 403.88, *p* < 0.001) significantly affect extraction yield. An extraction yield was profoundly influenced by the interaction between plant species and solvent polarity (*F* = 6.76, *p* = 0.0316), indicating that extraction efficiency varies with the plant matrix. *M. arcuata* had the highest water‐extractable matter at 36.27*%* ± 1.22*%*, whereas its ethanol‐extractable matter was 17.07*%* ± 2.60*%*. In contrast, *P. aquilinum* had lower solubility, with yields of 16.40*%* ± 0.40*%* in water and only 1.60*%* ± 0.40*%* in ethanol. Post hoc analysis using Tukey′s HSD confirmed significant differences in extraction yields between water and ethanol for both *M. arcuata* and *P. aquilinum* (Table 2).

**Table 2:** Physicochemical investigations of *Mussaenda arcuata* and *Pteridium aquilinum* plant species.

Plant species	Extraction solvents	*M* *e* *a* *n* *y* *i* *e* *l* *d* (*%*) ± *S* *D*
*Mussaenda arcuata*	Water	36.3 ± 1.22^a^
	Ethanol	17.1 ± 2.60^b^
*Pteridium aquilinum*	Water	16.4 ± 0.40^b^
	Ethanol	1.6 ± 0.40^c^

*Note:* *M* *e* *a* *n* ± *S* *D* (*n* = 3). Different superscript letters indicate statistical significance at the 95% confidence level (*p* < 0.05). Values sharing the letter “b” (*M. arcuata* ethanol and *P. aquilinum* water) were found to have no significant difference (*p* = 0.942), whereas all other pairwise comparisons were highly significant (*p* < 0.001).

### 3.3. Phytochemical Screenings

Qualitative phytochemical screening of *M. arcuata* and *P. aquilinum* revealed a diverse profile of secondary metabolites, confirming the presence of bioactive classes with therapeutic potential. Both species were positive for nitrogenous compounds (alkaloids) and various polyphenolic constituents, including flavonoids, phenols, and tannins. The consistent presence of surfactants (saponins) and lipid‐based compounds (steroids, terpenoids, and oils) was observed in both plant extracts. Besides, the primary metabolites like proteins and amino acids, along with secondary metabolites such as quinones and glycosides, were identified, which feature the intricate biochemical composition of these WEPs.

### 3.4. In Vitro Antibacterial Activity

The antibacterial evaluation of *M. arcuata* extracts demonstrated significant, dose‐dependent inhibitory effects on all bacterial species tested (Table 3). A three‐way ANOVA revealed that the solvent type, bacterial species, and extract concentration significantly influenced the ZOIs (*p* < 0.001), with a notable interaction among these factors, indicating varying solvent effectiveness based on bacterial strain and dose. The acetone extract exhibited greater antibacterial potency than the ethanol extract in all experiments. The highest antibacterial activity of the acetone extract against *S. aureus* was observed at 100 mg/mL, with a ZOI of 17.2 ± 0.11 mm (*p* < 0.05), significantly surpassing the ethanol extract′s maximum activity of 16.2 ± 0.07 mm at the same concentration. The potency of acetone at 75 mg/mL (16.2 ± 0.05^f^) is not statistically different from the potency of ethanol at its highest concentration (100 mg/mL, 16.2 ± 0.07^f^). Both extracts got their minimum effective potency at 25 mg/mL against *E. coli*, with no statistical difference between the two solvents (9.36 ± 0.092^a^vs.9.20 ± 0.02^a^). All concentrations of *P. aquilinum*, extracted with both solvents, exhibited no antibacterial activity against the studied bacterial strains. In contrast, gentamicin (10 *μ*g/mL) produced approximately 20 mm ZOIs for both species, whereas the plant extracts required significantly higher concentrations for similar efficacy.

**Table 3:** Antibacterial activity of acetone and ethanol extract (ZOI in millimeters) of *Mussaenda arcuata*.

Extract type	Bacteria	100 mg/mL	75 mg/mL	50 mg/mL	25 mg/mL	10 mg/mL	10 *μ*g/mL
Acetone	*S. aureus*	17.2 ± 0.11^h^	16.2 ± 0.05^f^	15.3 ± 0.115^e^	13.4 ± 0.10^b^	NA	NM
	*E. coli*	15.3 ± 0.11^e^	12.4 ± 0.083^c^	10.4 ± 0.242^d^	9.36 ± 0.09^a^	NA	NM
Ethanol	*S. aureus*	16.2 ± 0.07^f^	14.23 ± 0.10^g^	13.26 ± 0.08^b^	12.25 ± 0.12^c^	NA	NM
	*E. coli*	13.31 ± 0.15^b^	12.27 ± 0.06^c^	10.1 ± 0.12^d^	9.2 ± 0.02^a^	NA	NM
Gentamycin	*S. aureus*	NA	NA	NA	NA	NA	20.10 ± 0.12
	*E. coli*	NA	NA	NA	NA	NA	20.38 ± 0.16

*Note:* *M* *e* *a* *n* ± *S* *D* (*n* = 3). Superscript letters (a–h) represent groups from the three‐way ANOVA followed by Tukey′s HSD. Different letters indicate statistical significance at the 95% confidence level (*p* < 0.05, Tukey′s HSD). Values sharing the same superscript letter (a–h) are not significantly different (*p* > 0.05). Values sharing the same letter were not statistically significant.

Abbreviations: NA, not applicable; NM, not measurable.

Moreover, the antibacterial activity of ethanol and acetone extracts of *M. arcuata* and *P. aquilinum* was further assessed to determine MIC by observing bacterial colony growth on agar plates inoculated with *S. aureus* and *E. coli* at various extract concentrations (10–100 mg/mL). Growth outcomes were compared against positive (gentamicin, 10 *μ*g/mL) and negative (2% DMSO) controls. The summary of the results is presented in Table 4.

**Table 4:** Minimum inhibitory concentration of ethanol and acetone extract of *Mussaenda arcuata* using the broth dilution method.

Bacterial strain	Concentration (mg/mL)	Concentration percentage	Bacterial colony growth under test plant species		Bacterial colony growth under control substances	
			*Mussaenda arcuata*		Positive control	Negative control
			Ethanol extract	Acetone extract	Gentamicin (10 *μ*g/mL)	DMSO (2%)
*Staphylococcus aureus*	100	100%	−ve	−ve	−ve	+ve
	75	75%	−ve	−ve	−ve	+ve
	50	50%	−ve	−ve	−ve	+ve
	25	25%	−ve	−ve	−ve	+ve
	10	10%	NA	NA	NA	NA

*Escherichia coli*	100	100%	−ve	−ve	−ve	+ve
	75	75%	−ve	−ve	−ve	+ve
	50	50%	−ve	−ve	−ve	+ve
	25	25%	−ve	−ve	−ve	+ve
	10	10%	NA	NA	NA	NA

*Note:* −ve = no bacterial colony growth (antibacterial activity observed); +ve = there is bacterial colony growth (no antibacterial activity).

Abbreviation: NA, not assessed.

At all used concentrations from 25 to 100 mg/mL, both the ethanol and acetone extracts of *M. arcuata* exhibited complete bacterial colony growth inhibition against *S. aureus* and *E. coli* (labeled as “−ve” for inhibited colony growth). The MIC data was not available for the lowest concentration (10 mg/mL). On the other hand, neither the ethanol nor the acetone extracts of *P. aquilinum* were able to inhibit the bacterial growth at all used concentrations, indicating no antibacterial activity. Gentamicin successfully inhibited the growth of *S. aureus* and *E. coli.* There was no antibacterial activity in the negative control (2% DMSO), indicating that the solvent had no effect on bacterial growth. In summary, *M. arcuata* demonstrated notable antibacterial activities against both bacterial strains at the MIC of 25 mg/mL, whereas *P. aquilinum* extracts were ineffective under all the experimental conditions employed.

## 4. Discussion

WEPs have been utilized as a source of nutrition and health in rural Ethiopian communities, especially where healthcare and food diversity are limited, to tackle food insecurity and limited access to medical services [17, 89]. These plants are perceived as healthy alternatives to cultivated vegetables that might be rich in pesticides and chemicals [12]. They provide nutritional benefits and contain bioactive compounds like alkaloids, flavonoids, phenolics, and terpenoids, which possess medicinal properties, including anti‐inflammatory, antioxidant, antidiabetic, and antimicrobial effects [90, 91]. WEPs are gaining recognition in the pharmaceutical field for their antibacterial effects, which may potentially support addressing global health challenges like AMR, thereby extending their use beyond mere nutrition [92–94]. They are now being considered for possible novel antibacterial agents [95]. Thus, investigating the phytochemical composition and antibacterial activity of potential WEPs is essential for confirming their traditional uses and for discovering new natural compounds to develop alternative therapeutic agents.

The current study investigated the phytochemical composition and antibacterial properties of two selected WEPs, the *M. arcuata* and *P. aquilinum*, collected from southwestern Ethiopia. Phytochemical analysis plays a crucial role in plant defense mechanisms and has been utilized as a drug for thousands of years [96]. The qualitative phytochemical screening of the studied plants revealed the presence of various secondary metabolites, such as alkaloids, flavonoids, phenols, tannins, saponins, glycosides, and quinones. The joint study previously conducted reported the quantitative evaluation of bioactive compounds and revealed that *M. arcuata* has a high phenolic content of 468.79 mg GAE/100 g and notable flavonoid levels of 113.52 mg CE/100 g [97]. Although the presence of secondary metabolites does not guarantee substantial antibacterial activities, many of these compounds exhibit significant antimicrobial activity [98]. Besides, understanding their mechanisms of action is essential for the development of more potent antimicrobial medicines [99]. It has been evidenced that phenolic compounds, alkaloids, saponins, and terpenoids possess significant antibacterial properties, mainly through mechanisms such as membrane disruption, protein binding, interference with metabolism, antiquorum sensing, and antibiofilm activity [100].

The antibacterial activity of *M. arcuata* extracts showed a dose‐dependent inhibition against Gram‐positive (*S. aureus*) and Gram‐negative (*E. coli*) bacteria. The acetone extracts demonstrated higher potency than the ethanol extract, with a ZOI of 17.2 mm for acetone compared with 16.2 mm for ethanol at 100 mg/mL. Both extracts were able to inhibit the growth of *S. aureus* and *E. coli* at higher concentrations, with acetone proving more effective than ethanol in extracting various bioactive phytochemicals that enhance antibacterial activity. These antibacterial activity differences between acetone and ethanol extracts observed are likely due to variations in extraction efficiency, influenced by the polarity of the phytochemicals in plants. Acetone is a known solvent with intermediate polarity that effectively extracts various phytochemicals, including phenolics [101] and flavonoids [102], which are important for antimicrobial activity. Besides, the selection of an effective extraction process for medicinal plants relies on the phytochemical compounds that are responsible for the desired biological activity [103]. *S. aureus* is more susceptible to drugs than *E. coli*, likely due to differences in cell wall structures; *E. coli*, being Gram‐negative, has a thick lipopolysaccharide layer that reduces drug permeability and increases resistance [104].

Contrarily, the extracts of *P. aquilinum* exhibited no antibacterial activity against the tested strains, unlike *M. arcuata*. This could arise from three possible reasons: A low concentration of bioactive constituents in the plant, incompatible extraction methods, and environmental factors like altitude, soil type, and change of season could significantly affect phytochemical composition [105–108]. Besides, the bacterial strains utilized in this study might be inherently resistant to the bioactive compounds present in *P. aquilinum*, indicating a lack of potency or the need for higher concentrations than tested.

Efficacy at lower doses decreased significantly, with nonapplicability noted at 10 mg/mL. Gentamicin showed strong inhibition at much lower concentrations, suggesting that *M. arcuata* may need further purification to align with commercial antibiotics. Besides, determination of the MIC showed that ethanol and acetone extracts of *M. arcuata* completely inhibited colony growth of *S. aureus* and *E. coli* at concentrations of 25–100 mg/mL, with a definitive MIC established at 25 mg/mL. Similar MIC values for plant extracts against *S*. *aureus* and *E*. *coli* range from approximately 6.25 to 25 mg/mL, influenced by the plant species and extraction solvent used [109]. Despite these positive results, the extracts were less effective than conventional antibiotics, as gentamicin inhibited growth at just 10 *μ*g/mL. The negative control with 2% DMSO confirmed that the inhibition was due to plant metabolites.

The physicochemical evaluation indicated that *M. arcuata* had a higher extractable matter than *P. aquilinum* in both aqueous and ethanol solvents. This extractive value is a key pharmacognostic parameter that estimates soluble phytochemicals in plants [110]. Polar solvents effectively extract polar phytochemicals [111], and the higher aqueous extractive value for *M. arcuata* suggests a greater content of water‐soluble phytochemicals. Extraction yield differences are also influenced by solvent polarity, which affects the types and amounts of phytochemicals extracted.

In the current study, while the antibacterial potential and phytochemical composition of WEPs show promise, their application as nutraceuticals necessitates thorough toxicological validation. It has been reported that although the bioactive compounds of WEPs provide health benefits at low doses, high doses or prolonged use may result in adverse effects, such as organ‐specific toxicity and drug interactions [112]. Thus, a clear safety profile is essential before proposing it as a nutraceutical. Further study covering the standardized in vivo toxicity testing, specifically concerning long‐term liver and kidney function, is crucial to establish safe daily intake limits for humans.

Despite thoroughly examining physicochemical, phytochemical, and antibacterial properties of selected WEPs, the study had limitations, including a lack of specific controls in qualitative phytochemical screening, no quantitative analysis of bioactive compounds, unassessed antioxidant effects, and proximate composition. In vitro antibacterial assessments were restricted to two bacterial strains, and in vivo safety evaluations were not conducted, leading to a potentially imprecise broad MIC concentration range.

## 5. Conclusion

This study provides empirical evidence of the antibacterial activity potential of WEPs from southwestern Ethiopia, a promising area for future pharmacological exploration. *M. arcuata* fruit extracts demonstrate significant antibacterial activity against *S. aureus* and *E. coli*, with a ZOI between 9.20 ± 0.01 and 17.23 ± 0.06 mm. Phytochemical screening identified 13 bioactive compounds linked to this antibacterial efficacy, with acetone extracts shown to be more effective than ethanol. Besides, *M. arcuata* is suggested for further study on its active compounds and safety, enabling novel antimicrobial agents. Conversely, *P. aquilinum*, despite having bioactive compounds, did not show significant antibacterial effects. Future research should focus on isolation and characterization of phytochemicals in WEPs and use controls for accuracy, as *P. aquilinum* showed no significant antibacterial effects despite its bioactive compounds. In vivo studies are necessary to assess safety and efficacy, along with a systematic evaluation of additional WEPs. Evidence‐based guidelines for the sustainable use and conservation of significant species like *M. arcuata* are also recommended.

**Nomenclature**
AHRI: Armauer Hansen Research Institute
AMR: antimicrobial resistance
ANOVA: analysis of variance
ATCC: American Type Culture Collection
CCNP: Chebera Churchura National Park
DMSO: dimethyl sulfoxide
*E. coli*
: *Escherichia coli*
*M. arcuata*
: *Mussaenda arcuata*
MCDA: multicriteria decision analysis
MHA: Mueller–Hinton agar
MIC: minimum inhibitory concentration
*P. aquilinum*
: *Pteridium aquilinum*
*S. aureus*
: *Staphylococcus aureus*
SD: standard deviation
WEPs: wild edible plants
WHO: World Health Organization
ZOI: zone of inhibition

## Funding

No funding was received for this research.

## Ethics Statement

An approval letter for this study was granted by the Institutional Review Board of the Jimma University Institute of Health and the postgraduate director, with Reference No. JUIH/IRB/259/24, after its ethical issue was reviewed. The ethical clearance letter was used to get permission for data collection from the study areas. The questionnaires were translated from the English version to the local languages of the study areas. Participants participated voluntarily, with the right to decline participation in the study. Confidentiality and privacy of the information were assured using an anonymous questionnaire and maintained in a secure place.

## Conflicts of Interest

The authors declare no conflicts of interest.

## Supporting information


**Supporting Information** Additional supporting information can be found online in the Supporting Information section. File S1: Additional data regarding the consent form, semistructured questionnaire, and the checklist of focus group discussion and approval letter.

## Data Availability

The data that support the findings of this study are available from the corresponding authors upon reasonable request.
