# Supplementary material for: Phytochemical and In Vitro Antibacterial Assessment of Selected Wild Edible Plants From Southwestern Ethiopia
Source: Int J Food Sci. 2026 Jul 9;2026:5113561. doi: 10.1155/ijfo/5113561 (PMC13348544; doi:10.1155/ijfo/5113561)
Supplement: Supplementary file 1 — Supporting Information Additional supporting information can be found online in the Supporting Information section. File S1: Additional data regarding the consent form, semistructured questionnaire, and the checklist of focus group discussion and approval letter. [file IJFO-2026-5113561-s001.docx]

**Multi-Criteria Decision Analysis**

Table 1 presents in the manuscript a comprehensive evaluation of the 22 medicinally useful WEPs identified by ethnobotanical surveys, based on a predesigned set of ten selection criteria. Each plant species was evaluated based on these ten criteria designed to ensure scientific rigor, practical applicability, and ethnobotanical relevance. The criteria included completeness of ethnobotanical data, the possibility to collect plant parts in bulk, accessibility of plant species within study areas, availability of plant species during the data collection period, appropriateness of the plant’s size for collection, usage in treating major health problems in Ethiopia, taxonomic identifiability of plant species by experts, ease of harvesting under various climatic conditions, indigenousness of plant species to Ethiopia, and novelty of plant species (whether the plant parts have not been studied previously).

Each WEP species was scored per criterion with a binary value, where 1 meant the species met the criterion and 0 meant it did not meet the criterion. The overall scores of all evaluated plant species ranged from 4 to 9, enabling systematic ranking for candidate selection.

Among the listed WEPs, *M. arcuata* and *P. aquilinum* achieved the highest total score of 9, meeting nearly all the criteria effectively. Notably, *M. arcuata* has an additional advantage because its part (fruit) has not been previously investigated scientifically. This enhances its potential to contribute to new ethnopharmacological insights. *Acanthus sennii, Ajuga integrifolia, Celosia trigyna L., Lippia adoensis Hochst., Rumex nervosus Vahl, and Sporobolus pyramidalis* were WEPs scoring the second-highest total scores of 8*.* Despite not being novel and indigenous, these plant species showed encouraging traits like accessibility, medicinal use, and ease of harvesting.

Conversely, WEP species such as *Dovyalis abyssinica, Cordia africana Lam,* and *Phoenix reclinate* received the lowest scores (4 points each). This indicates that they were less suitable for the current research, principally due to limited availability during data collection, previous study saturation, and restricted medical use.

In summary, high-scoring WEPs demonstrated significant potential for further investigation. Consequently, the two WEPs that were selected as promising species for further laboratory investigations were *M. arcuata* and *P. aquilinum.*

Table 1: Multi-criteria decision analysis approach used to select wild edible plants for experimental studies

| **S.No** | **Identified WEPs (Scientific name)** | **Selection Criteria** | | | | | | | | | | |
| --- | --- | --- | --- | --- | --- | --- | --- | --- | --- | --- | --- | --- |
|  |  | **Plant species with complete data** | **Accessible plant species in study areas** | **Plant parts can be collected in bulk** | **Plant species available at the time of data collection** | **Plant species appropriate for collection (small in size)** | **Taxonomically identifiable plant species by experts** | **Easily harvestable plant species in various climates** | **Plants used to treat major health problems in Ethiopia** | **Indigenous plant species to Ethiopia** | **Novel plant part** | **Total Score** |
|  | *Ajuga integrifolia* | 1 | 1 | 1 | 1 | 1 | 1 | 1 | 1 | 0 | 0 | **8** |
|  | *Pteridium aquilinum* | 1 | 1 | 1 | 1 | 1 | 1 | 1 | 1 | 0 | 1 | **9** |
|  | *Celosia trigyna L* | 1 | 1 | 1 | 1 | 1 | 1 | 1 | 1 | 0 | 0 | **8** |
|  | *Rumex nervosus Vahl* | 1 | 1 | 1 | 1 | 1 | 1 | 1 | 1 | 0 | 0 | **8** |
|  | *Kalanchoe petitiana A. Rich* | 1 | 1 | 0 | 1 | 1 | 1 | 1 | 1 | 0 | 0 | **7** |
|  | *Solanum nigrum L.* | 1 | 1 | 1 | 1 | 1 | 1 | 1 | 0 | 0 | 0 | **7** |
|  | *Embelia schimperi Vatke* | 1 | 1 | 1 | 1 | 0 | 1 | 0 | 1 | 0 | 0 | **6** |
|  | *Carissa spinarum (Vahl.) Forssk. Ex Endl.* | 1 | 1 | 1 | 1 | 1 | 1 | 0 | 1 | 0 | 0 | **7** |
|  | *Punica granatum* | 1 | 1 | 0 | 0 | 0 | 1 | 1 | 1 | 0 | 0 | **5** |
|  | *Croton macrostachyus Hochst.* | 1 | 1 | 1 | 1 | 0 | 1 | 0 | 1 | 0 | 0 | **6** |
|  | *Impatiens rothii Hook. f* | 1 | 1 | 0 | 0 | 1 | 1 | 1 | 1 | 0 | 1 | **7** |
|  | *Rubus apetalus* | 1 | 1 | 0 | 1 | 0 | 1 | 0 | 1 | 0 | 0 | **5** |
|  | *Rosa abyssinica L.* | 1 | 1 | 1 | 1 | 0 | 1 | 1 | 1 | 0 | 0 | **7** |
|  | *Physalis peruviana L* | 1 | 1 | 0 | 1 | 1 | 1 | 1 | 0 | 0 | 0 | **6** |
|  | *Syzygium guineense* | 1 | 1 | 1 | 1 | 0 | 1 | 0 | 1 | 0 | 0 | **6** |
|  | *Cordia africana Lam* | 1 | 1 | 1 | 0 | 0 | 1 | 0 | 0 | 0 | 0 | **4** |
|  | *Phoenix reclinate* | 1 | 1 | 0 | 0 | 0 | 1 | 0 | 0 | 0 | 1 | **4** |
|  | *Dovyalis abyssinica* | 1 | 1 | 0 | 1 | 0 | 1 | 0 | 0 | 0 | 0 | **4** |
|  | *Lippia adoensis Hochst.* | 1 | 1 | 0 | 1 | 1 | 1 | 1 | 1 | 1 | 0 | **8** |
|  | *Acanthus sennii* | 1 | 1 | 1 | 1 | 1 | 1 | 1 | 0 | 1 | 0 | **8** |
|  | *Mussaenda arcuata* | 1 | 1 | 1 | 1 | 1 | 1 | 1 | 1 | 0 | 1 | **9** |
|  | *Sporobolus pyramidalis P* | 1 | 1 | 0 | 1 | 1 | 1 | 1 | 1 | 0 | 1 | **8** |

| ***Mussaenda arcuata*** | |
| --- | --- |
| 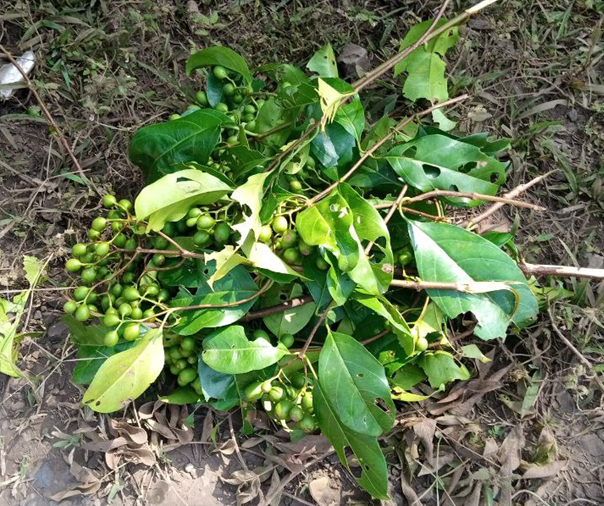 | 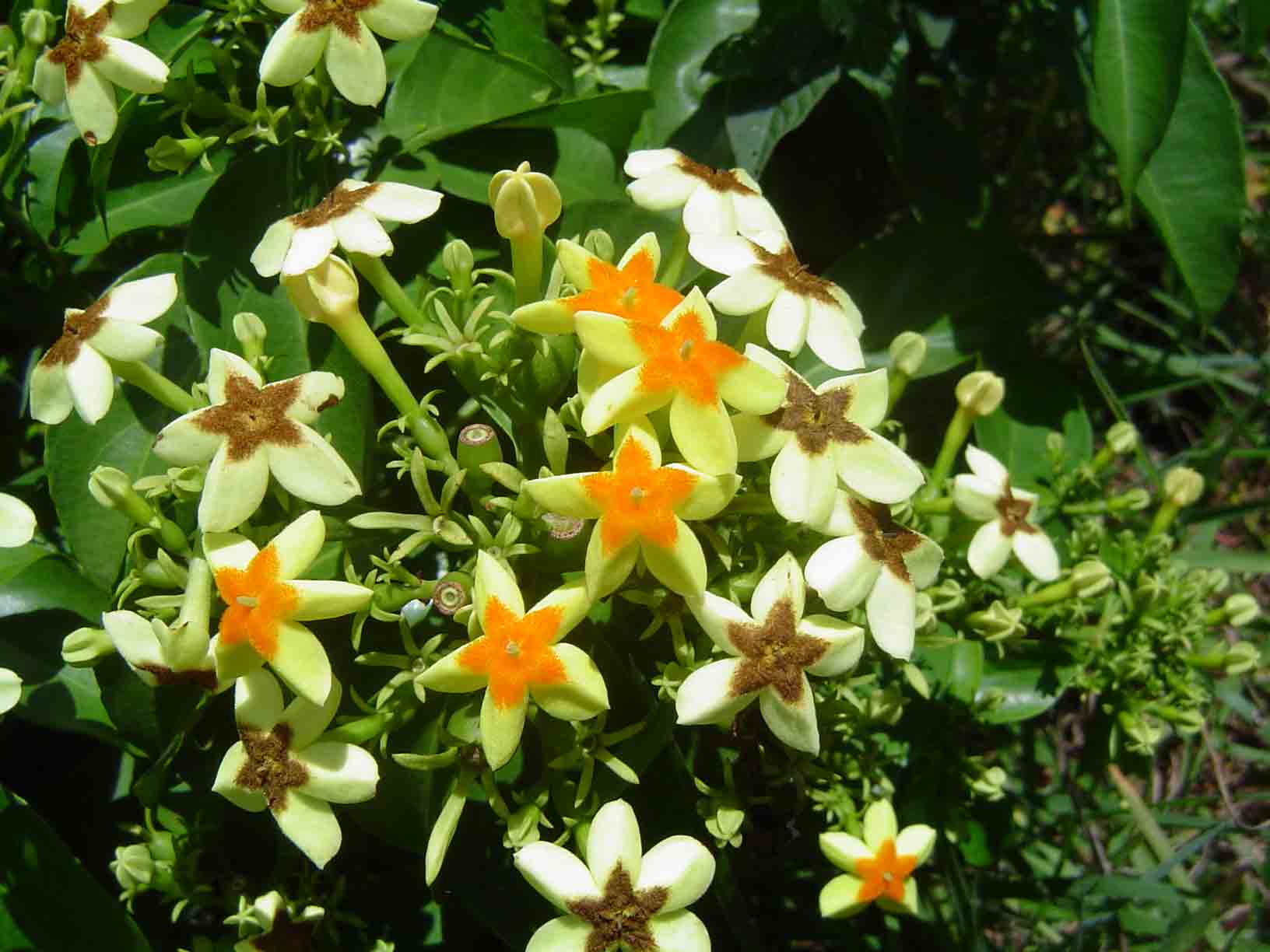 |

| ***Pteridium aquilinum*** | |
| --- | --- |
|  |  |

**Figure S1: Images of selected wild edible plant species**
